# Generation of selenium hydride from alkaline solutions: a new concept of the hydride generation-atomic absorption technique

**DOI:** 10.1155/S1463924689000337

**Published:** 1989

**Authors:** Ragnar Bye

**Affiliations:** Department of Chemistry Agricultural University of Norway Box 30 1432 Aas-NLH Norway

## Abstract

The use of hydride generation is often useful in environmental analysis. The normal acid sodium tetrahydroborate reaction provides exceptional sensitivity with continuous flow hydride generators. In some situations there are interferences which will mask the sensitivity. An alternative chemistry system is described here and is shown to offer similar sensitivity to that normally used. A commercial continuous flow analyser is used in this work.


Journal of Automatic Chemistry Vol. 11, No. 4 (July–August 1989), pp. 156–158

### Generation of selenium hydride from alkaline solutions: a new concept of the hydride generation–atomic absorption technique

**Ragnar Bye**
*Department of Chemistry, Agricultural University of Norway, Box 30, 1432 Aas-NLH, Norway*

*The use of hydride generation is often useful in environmental analysis. The normal acid sodium tetrahydroborate reaction provides exceptional sensitivity with continuous flow hydride generators. In some situations there are interferences which will mask the sensitivity. An alternative chemistry system is described here and is shown to offer similar sensitivity to that normally used. A commercial continuous flow analyser is used in this work.*

### Introduction

Hydride generation–atomic absorption spectrometry (HGAAS) has gained wide acceptance as a versatile technique for the determination of a number of elements, i.e. the so-called hydride-forming elements that include arsenic, antimony, bismuth, germanium, lead, selenium, tellurium and tin [1–3].

The principle of the method involves a reduction of the element from a higher oxidation state to its lowest state (usually −II or −III), which appears as the volatile hydride. This in turn is flushed from the sample solution by an inert gas (usually argon) and into the atom cell. The cell is heated either electrically or by a flame in order to atomize the element. Sodium tetrahydroborate(III) is now almost exclusively used as reductant. The design and the operation principle of the atom cell may vary: inert gas – hydrogen diffusion flames, graphite furnaces and quartz tubes that may be externally heated or are of the flame-in-tube type. The present status is that the quartz tube technique is the predominating one.

For an effective release of the gaseous hydrides some parameters are of importance: the sample acidity, the concentration and the flow of reducing agent (tetrahydroborate). The demand for an acidic sample solution is demonstrated by the following equations for selenium:

$$H_2Se_{(g)} \overset{H_2O}{\rightleftharpoons} H_2Se_{(aq)}$$

$$H_2Se_{(aq)} + H_2O \rightleftharpoons H_3O^+ + HSe^-_{(aq)} \qquad K_{a_1} = 1{\cdot}3 \times 10^{-4}$$

$$HSe^-_{(aq)} + H_2O \rightleftharpoons H_3O^+ + Se^{2-}_{(aq)} \qquad K_{a_2} = 1{\cdot}0 \times 10^{-11}$$

However the demand for acidic sample solutions for an effective generation of the hydrides has so far been a limitation of the technique. Obviously there are cases when it is desirable to have the possibility of analysing alkaline samples without having to make them acidic *before* the analysis. One reason for this is that the chemical form of an element (that is, the oxidation state) may alter as a result of acidification. For example, sea water may contain both Se(VI) and Se(IV). It is well known that Se(VI) is not reducible by tetrahydroborate. This implies that it is possible to distinguish between Se(VI) and Se(IV) in such samples, because Se(IV) can be determined separately and, after a chemical reduction of Se(VI) to Se(IV), the total concentration of selenium (that is Se(IV) + Se(VI)) can be determined. Chloride ions in acid solutions serve as a very convenient reductant of Se(VI) to Se(IV) [4]. However, if a sample is acidified *before* the analysis (for instance for preservation purposes), this reaction is likely to take place *within* the time of analysis, prohibiting the possibility of determining both oxidation forms.

Another advantage of hydride generation from alkaline solutions is the possibility of simultaneous removal of seriously interfering elements such as nickel and copper as the hydroxides [5]. This will be described in a subsequent paper. In this paper the principle of hydride generation from alkaline solutions using an automatic hydride generator is described and the results of the test experiments are given.

### Experimental

### *Apparatus*

A Perkin-Elmer Model 300 atomic absorption spectrometer equipped with an electrodeless discharge lamp for selenium and operated at the 196·0 nm resonance line was used. An automatic hydride generator, P.S. Analytical (U.K.), was operated with the following settings: delay 15, measure 30 and reset 20 sec. The flow of the reagent streams were changed as described in the text below.

### *Reagents*

Sodium tetrahydroborate(III) solution (2% m/v) was prepared by dissolving $NaBH_4$ (pro analysi, Fluka) in a sodium hydroxide solution (1% m/v, pro analysi, Merck). The solution was filtered before use. Hydrochloric acid was of analytical grade (Merck). Selenium(IV) standard solutions were prepared by

0142–0453/89 $3.00 © 1989 Taylor & Francis Ltd.

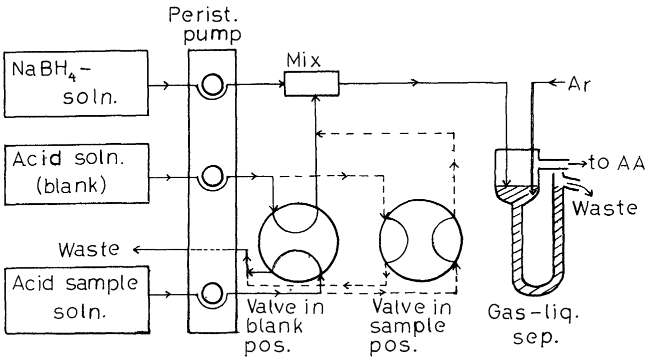

*Figure 1. Flow diagram of the automatic hydride generator used in the conventional way (for acid solutions).*

diluting a 4 g $l^{-1}$ Se(IV) solution made by dissolving 13·323 g of $Na_2SeO_3{\cdot}5H_2O$ (pro analysi, Fluka) in water and filled to 1 litre.

*Hydride generation frrom alkaline solutions*

The principle of hydride generation from alkaline solutions using an automatic system is shown in figure 2. This should be compared with figure 1 which illustrates the flow scheme for the normal method of hydride generation (i.e. from acid solutions). In the 'alkaline' modification, the tetrahydroborate and the acid solutions are continuously mixed in the 'blank' mode. In the 'sample' mode the alkaline sample solution (containing $NaBH_4$) is mixed with hydrochloric acid. The generated hydride is separated in the gas–liquid separator in the usual way, and is flushed into the flame-heated quartz cell in the spectrometer.

Thus, the alteration of the set-up simply implies an interchange of the tetrahydroborate and the hydrochloric acid streams; the former solution now acting as a 'blank' instead of the hydrochloric acid solution.

*Procedure*

To a suitable volume of the aqueous test solution in a 100 ml volumetric flask, 5 ml of 1·0 mol $l^{-1}$ NaOH solution was added (pH 12–13). 10 ml of the $NaBH_4$ solution was

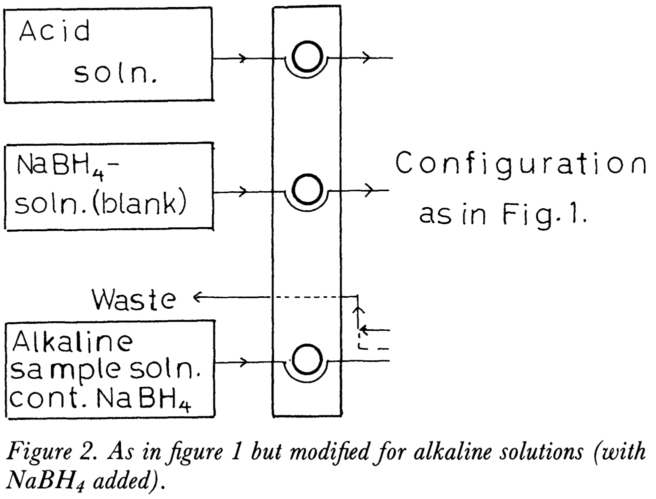

*Figure 2. As in figure 1 but modified for alkaline solutions (with $NaBH_4$ added).*

added and the solution was filled to volume and mixed well. The solutions were analysed for Se(IV) using the automatic hydride generation technique. Each result given is the mean of three measurements.

*Testing of the method*

Two series of selenium solutions were made: one series contained 0, 10, 20 and 30 μg $l^{-1}$ of Se(IV) in 4·0 mol $l^{-1}$ HCl, and the other contained the same concentrations of Se(IV) in a $NaOH/NaBH_4$ solution as described in the *Procedure.*

In order to examine which concentration of hydrochloric acid was the optimal for the transformation of selenide to selenium hydride and for the release of the selenium hydride gas, a number of concentrations of HCl were tested: 4, 6, 8, and 10 mol $l^{-1}$.

### Results and Discussion

The 'acid' (the normal) series was made in order to compare the sensitivity of results obtained from the alkaline solutions with those obtained in the normal manner.

In the alkaline solutions the selenium will be present as selenide ions ($Se^{2-}$) after the addition of tetrahydroborate. When the solutions are mixed with hydrochloric acid in the T-piece mixing chamber, gaseous $H_2Se$ is immediately formed according to the equations given in the *Introduction.* The efficiency of this formation is apparently dependent on the concentration of the hydrochloric acid as shown in figure 3, which demonstrates that the highest concentration of HCl (10 mol $l^{-1}$)

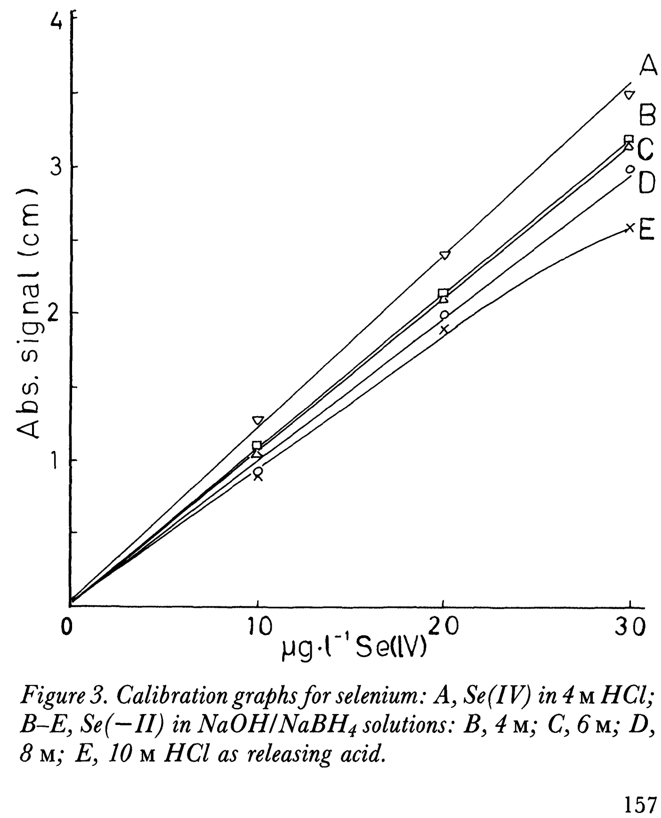

*Figure 3. Calibration graphs for selenium: A, Se(IV) in 4* M *HCl; B–E, Se(−II) in $NaOH/NaBH_4$ solutions: B, 4* M*; C, 6* M*; D, 8* M*; E, 10* M *HCl as releasing acid.*

resulted in a slight curvature of the standard graph, whereas straight lines were obtained for the other concentrations of acid. The best sensitivity was obtained using 4 mol $l^{-1}$ of HCl, being about 92% of the signals from the hydrochloric acid (the normal) test solutions.

These results clearly indicate that the alternate procedure described here will provide similar sensitivity to the normal acid conditions used. The advantages of the akaline chemistry described in the *Introduction* to this paper can now be exploited in analytical applications.

### References

1. Godden, R. G. and Thomerson, D. R., *Analyst,* **105** (1980), 1137.

2. Nakahara, T., *Progress Anal. At. Spectr.,* **6** (1983), 163.

3. Dedina, J., *Progress Analyt. Spectr.,* **11** (1988), 251.

4. Bye, R. and Lund, W., *Fresenius Z. Anal. Chem.,* **332** (1988), 242.

5. Welz, B. and Melcher, M., *Anal. Chimica Acta,* **153** (1983), 297.

